# Antibiofilm Activity of Heather and Manuka Honeys and Antivirulence Potential of Some of Their Constituents on the DsbA1 Enzyme of *Pseudomonas aeruginosa*

**DOI:** 10.3390/antibiotics9120911

**Published:** 2020-12-15

**Authors:** Oscar Shirlaw, Zara Billah, Baraa Attar, Lisa Hughes, Rana M. Qasaymeh, Veronique Seidel, Georgios Efthimiou

**Affiliations:** 1 Strathclyde Institute of Pharmacy and Biomedical Sciences, University of Strathclyde, Glasgow G4 0RE, UK; oscar.shirlaw.2016@uni.strath.ac.uk (O.S.); zara_billah@hotmail.co.uk (Z.B.); battar@me.com (B.A.); lisahughes6217@gmail.com (L.H.); rana-mohammad-mahmoud-qasaymeh@strath.ac.uk (R.M.Q.); 2 Department of Biomedical and Forensic Sciences, Hardy Building, University of Hull, Hull HU6 7RX, UK

**Keywords:** antibiofilm, antivirulence, Heather honey, Manuka honey, *Pseudomonas aeruginosa*

## Abstract

Heather honey was tested for its effect on the formation of biofilms by *Staphylococcus aureus*, *Pseudomonas aeruginosa*, *Escherichia coli*, *Klebsiella pneumoniae*, *Enterococcus faecalis*, *Salmonella Enteriditis* and *Acinetobacter baumanii* in comparison with Manuka honey. At 0.25 mg/mL, Heather honey inhibited biofilm formation in *S. aureus*, *A. baumanii*, *E. coli*, *S. Enteriditis* and *P. aeruginosa*, but promoted the growth of *E. faecalis* and *K. pneumoniae* biofilms. Manuka honey inhibited biofilm formation in *K. pneumoniae*, *E. faecalis*, and *S. Enteriditis*, *A. baumanii*, *E. coli* and *P. aeruginosa*, but promoted *S. aureus* biofilm formation. Molecular docking with Autodock Vina was performed to calculate the predictive binding affinities and ligand efficiencies of Manuka and Heather honey constituents for PaDsbA1, the main enzyme controlling the correct folding of virulence proteins in *Pseudomonas aeruginosa*. A number of constituents, including benzoic acid and methylglyoxal, present in Heather and/or Manuka honey, revealed high ligand efficiencies for the target enzyme. This helps support, to some extent, the decrease in *P. aeruginosa* biofilm formation observed for such honeys.

## 1. Introduction

Antimicrobial drug resistance, particularly in Gram-negative bacteria, is an ever-increasing challenge for healthcare systems worldwide [1]. Alternative treatment options to conventional antibiotics are urgently needed to tackle this global threat [2,3]. This includes the discovery of molecules that could disrupt the ability of pathogens to produce virulence factors [4,5]. In Gram-negative bacteria, various virulence factors are produced under the control of a master virulence regulatory oxidoreductase enzyme called DsbA. The latter catalyses the formation of disulfide bonds in proteins and, in doing so, is instrumental to the process of correct protein folding of bacterial virulence proteins, including type-IV fimbriae, flagellae and adhesion factors that play a central role in biofilm formation [6,7,8,9,10,11]. The disulfide bond forming pathways in Gram-positive bacteria are less well established [12].

Biofilms are formed when microbial communities, held together by a polymeric matrix, attach to surfaces. The formation of microbial biofilms poses significant risks in healthcare settings when pathogens attach to wounds, surfaces, and medical devices [13,14]. The opportunistic nosocomial pathogen, *Pseudomonas aeruginosa*, is one of the most common bacteria isolated from chronic wounds and has become difficult to eradicate due to its ability to form biofilms [15,16,17,18]. Its DsbA enzyme is an attractive target in the search for new antivirulence agents [19,20].

Products from the beehive have a long history of use as traditional remedies [21], and honey has emerged as a promising topical antibacterial agent [22,23,24,25,26]. Honey is known to contain a diversity of chemicals which vary depending on nectar sources in different geographical locations, harvesting seasons, types of bees foraging and storage of the final product [27,28,29]. The antimicrobial activity of most honeys has been attributed to a high sugar content, low pH, and the ability to produce hydrogen peroxide [22,30]. Other contributors to this activity include plant- and bee-derived chemicals [30]. Both Manuka honey (derived from the nectar of *Leptospermum* spp.) and Heather honey (from *Erica* spp.) have a similar pH, low hydrogen peroxide activity and a high sugar content [31] and contain various phytochemicals such as phenolic acids and flavonoids [32,33,34,35]. Manuka honey is very rich in methylglyoxal (MGO), a plant-derived compound formed during storage and used for ‘Unique Manuka Factor’ (UMF) grading [28,29,30,35,36]. Heather honey, on the other hand, is rich in abscisic acid [31,37].

Both honeys have demonstrated antibacterial activity [36,38,39] and an inhibitory effect on polymicrobial biofilms [40]. Manuka honey can disrupt biofilm formation in several pathogens [41,42,43,44,45,46,47,48,49]. To the best of our knowledge, the effect of Heather honey on monobacterial biofilms has never been reported, and neither has the potential affinity of Manuka nor Heather honey constituents for DsbA been predicted. Here, we compared the effect of Heather honey with that of Manuka honey on the formation of biofilms in seven bacteria. We also employed a molecular docking approach to predict the binding affinity of constituents from both honeys towards the *P. aeruginosa* DsbA enzyme (*Pa*DsbA1).

## 2. Results

### 2.1. Determination of the Time Required for Optimal Biofilm Formation

Optimal biofilm formation (OD550 nm 0.8–1.7) by all bacterial species was obtained after a 24 h incubation period. This time point was selected to further study biofilm formation in subsequent experiments. High amounts of biofilm were also observed after 48 h (OD550 nm 0.7–1.6), less so after 72 and 96 h (Supplementary Materials Figure S1).

### 2.2. Antibiofilm Activity

**Figure 1:**
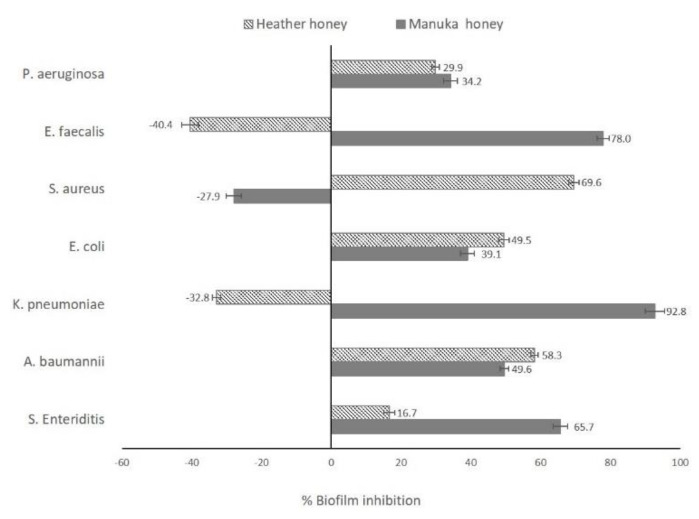
Inhibition of bacterial biofilm formation by Heather and Manuka honeys (0.25 mg/mL).

**Figure 2:**
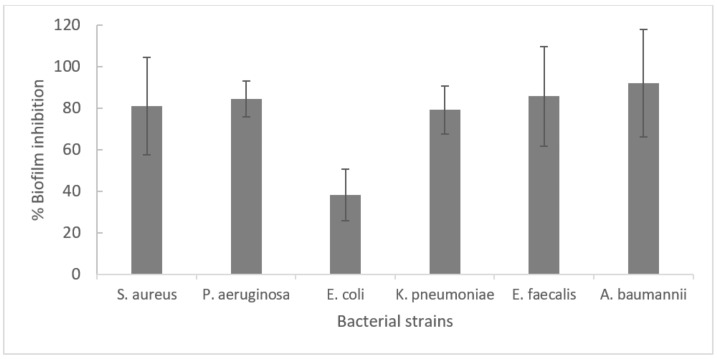
Inhibition of biofilm formation by oleanolic acid (OA) tested at a concentration of 0.0625 mg/mL.

At 0.25 mg/mL, Manuka honey showed the strongest inhibition of biofilm formation in *K. pneumoniae, E. faecalis*, and *S. Enteriditis* (92.8, 78.0, and 65.7% inhibition, respectively). It also inhibited biofilm formation in *A. baumanii*, *E. coli* and *P. aeruginosa*. Unlike Manuka honey, which increased *S. aureus* biofilm formation, Heather honey inhibited biofilm formation in *S. aureus* (69.6%). It decreased biofilm formation in *A. baumanii*, *E. coli, S. Enteriditis* and *P. aeruginosa,* but promoted the growth of *E. faecalis* biofilms (Figure 1). Oleanolic acid decreased biofilm formation in all bacteria (≥50% in all cases except for *E. coli*) (Figure 2).

### 2.3. Molecular Docking of Honey Constituents against P. aeruginosa DsbA1

**Figure 3:**
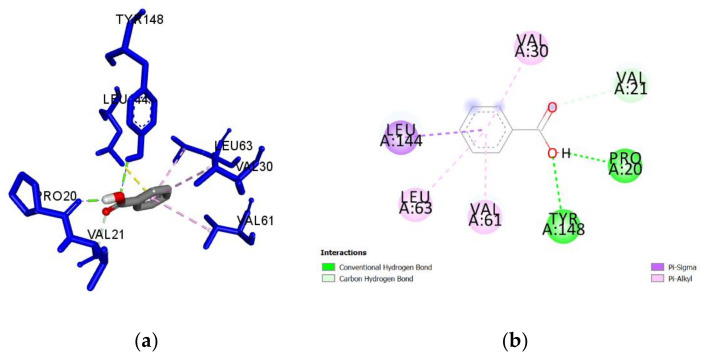
(**a**) Docked pose of benzoic acid in the *Pa*DsbA1 binding site showing molecular interactions—hydrogen and hydrophobic bonds shown as green and pink/purple dashed lines, respectively; (**b**) 2D plot of interactions between benzoic acid and key residues of *Pa*DsbA1 generated by BIOVIA Discovery Studio visualizer.

**Figure 4:**
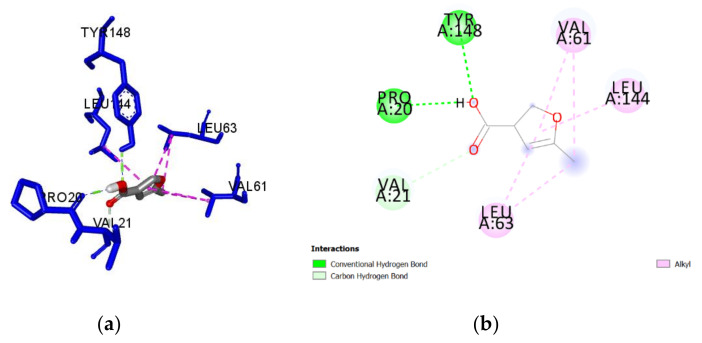
(**a**) Docked pose of 5-methyl-3-furancarboxylic acid in the *Pa*DsbA1 binding site showing molecular interactions—hydrogen and hydrophobic bonds shown as green and pink/purple dashed lines, respectively; (**b**) 2D plot of interactions between 5-methyl-3-furancarboxylic acid and key residues of *Pa*DsbA1 generated by BIOVIA Discovery Studio visualizer.

**Figure 5:**
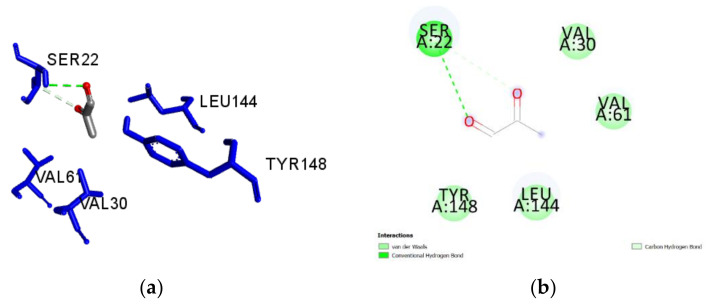
(**a**) Docked pose of methylglyoxal in the *Pa*DsbA1 binding site showing molecular interactions—hydrogen and hydrophobic bonds shown as green and pink/purple dashed lines, respectively; (**b**) 2D plot of interactions between methylglyoxal and key residues of *Pa*DsbA1 generated by BIOVIA Discovery Studio visualizer.

**Figure 6:**
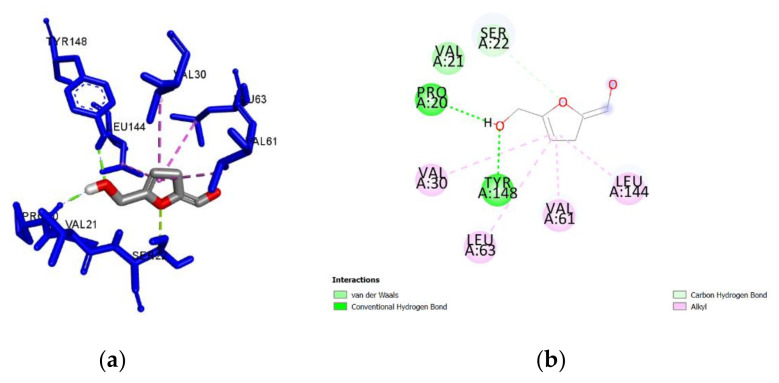
(**a**) Docked pose of 5-hydroxymethyl-2-furaldehyde in the *Pa*DsbA1 binding site showing molecular interactions—hydrogen and hydrophobic bonds shown as green and pink/purple dashed lines, respectively; (**b**) 2D plot of interactions between 5-hydroxymethyl-2-furaldehyde and key residues of *Pa*DsbA1 generated by BIOVIA Discovery Studio visualizer.

A guided docking approach was used to predict the binding affinities and ligand efficiency indices of 56 constituents of Manuka and Heather honey towards *Pa*DsbA1 (Supplementary Materials Table S1). The best ligand efficiencies were obtained for benzoic acid (0.60), 5-methyl-3-furancarboxylic acid (0.57), methylglyoxal (0.56) and 5-hydroxymethyl-2-furaldehyde (0.56) (Table 1). The molecular interactions of each of these ligands with *Pa*DsbA1 are detailed in Table 1 and in Figure 3, Figure 4, Figure 5 and Figure 6. Benzoic acid strongly interacted with *Pa*DsbA1 via two hydrogen bonds (contact distances < 2.5 Å) to Tyr148 and Pro20 and hydrophobic bonds with Leu144, Leu63, Val30, and Val61.

## 3. Discussion

Heather honey has been the subject of a limited number of studies investigating its effect on bacterial biofilms. Only one study to date has reported its inhibitory activity on mixed *Candida–Pseudomonas* biofilms [40]. This prompted us to test its activity against a range of Gram-positive and Gram-negative single-species biofilms. This was done using a crystal violet assay, a commonly-used indirect method of biofilm quantification [50], and in comparison with Manuka honey, already known for its ability to decrease bacterial biofilm formation [41,42,43,44,45,46,47,48,49]. The decrease in *S. aureus*, *A. baumanii*, *E. coli*, *S. Enteriditis* and *P. aeruginosa* biofilm formation observed for Heather honey is reported here for the first time. Manuka honey decreased biofilm formation in *P. aeruginosa*, *E. faecalis*, *A. baumanii*, and *E. coli*, in agreement with previous studies [45,46,47,48,49]. Its effect on *K. pneumoniae* and *S. Enteriditis* biofilms has not been previously reported. Heather and Manuka honey, both tested at sub-inhibitory concentrations, increased biofilm formation in the Gram-positive bacteria *E. faecalis* and *S. aureus*, respectively. Such an effect has been observed in other investigations [29,51]. It has been suggested that the decrease in biofilm formation reported for Manuka honey could be linked to the presence of phenolic compounds, bee defensin-1 and/or MGO. The latter is able to inhibit biofilm formation by altering the structures of bacterial fimbriae and flagellae [29,36]. Oleanolic acid is known to decrease biofilm formation [52,53,54,55], but this is the first report of such an effect against *E. faecalis*, *S. aureus*, *E. coli*, *K. pneumoniae*, *A. baumanii* and *S. Enteriditis* biofilms.

The DsbA enzymes of Gram-negative bacteria have emerged as attractive targets for the discovery and development of new antivirulence agents including from natural sources [56,57]. There are two main structural classes of DsbA enzymes, DsbA-I and DsbA-II. Both classes contain proteins that share highly conserved residues in their catalytic active sites, and also several identical hydrophobic amino acids adjacent to this site. DsbA-I proteins have a unique groove on their non-catalytic face, opposite to the active site surface. *Pa*DsbA1 was identified as the main DsbA-I type enzyme in *P. aeruginosa* [20]. Our molecular docking study was conducted to predict the binding affinity of honey constituents for the groove on the non-catalytic face the *Pa*DsbA1 [11,20].

Our predictive in silico analysis revealed that benzoic acid, a constituent of both Heather [58] and Manuka honey [59], could bind with a high efficiency into the pocket of *Pa*DsbA1 where the control ligand fits [20]. This binding occurs via H-bond interactions between the hydroxyl hydrogen of benzoic acid and the carbonyl oxygen of Pro20, and between the hydroxyl oxygen of benzoic acid and the phenolic hydrogen of Tyr148. Interestingly, previous studies reported that benzoic acid could attenuate the virulence of *P. aeruginosa* in plants and nematodes through inhibiting the production of virulence factors such as pyocyanin, and reducing total protease and elastase activity [60]. The latter exoenzyme plays a critical role in the virulence of *P. aeruginosa* and requires *Pa*DsbA1 for its biogenesis [6,10,20]. In the case of Manuka honey, our predictions also revealed that MGO had a high binding efficiency for *Pa*DsbA1. This could support the decrease in *P. aeruginosa* biofilm formation for this type of honey via alteration of fimbriae and flagellae. Further work is required to confirm this hypothesis and test MGO and other honey constituents showing high predictive binding efficiencies in our in silico screening for their ability to prevent biofilms in wild type bacteria and ΔDsbA mutants.

## 4. Materials and Methods

### 4.1. Bacterial Strains, Culture Conditions, and Inoculum Preparation

*Staphylococcus aureus* (ATCC 43300), *Pseudomonas aeruginosa* (ATCC 27853), *Escherichia coli* (ATCC 25922), *Klebsiella pneumoniae* (ATCC 700603), *Enterococcus faecalis* (ATCC 51299) and *Acinetobacter baumanii* (ATCC 19606) were obtained from the American Type Culture Collection. *Salmonella enteriditis* (NCTC 4444) was obtained from the National Collection of Type Cultures. In preparation for antibiofilm activity screening, each strain was grown in tryptone soya broth (TSB; Oxoid, UK) at 37 °C for 4 h (late exponential phase) under continuous shaking (250 rpm).

### 4.2. Honey Sources and Preparation of Samples

Heather honey was kindly provided by the Scottish Bee Company. Manuka honey (UMF 10+) was obtained from Wilkin & Sons Ltd., Tiptree, UK. An exactly weighed amount (around 1 g) of each sample was dissolved in water and then filter-sterilised (0.22 μm disks, Sartorius, UK) to afford stock solutions of Heather honey and Manuka honey (100 mg/mL).

### 4.3. Antibiofilm Assay

This was performed according to a previous method with some modifications [61]. Briefly, 100 μL of each bacterial inoculum (2.5 × 10^5^ CFU/mL) was added to the wells of a 24-flat well polystyrene plate, containing TSB (1.8 mL). Heather and Manuka honeys were tested at 0.25 mg/mL. Wells containing TSB only were used as the sterile controls. Oleanolic acid (Sigma-Aldrich, Gillingham, UK), a plant-derived triterpenoid which inhibits the formation of biofilms in Gram-positive and Gram-negative bacteria, was used as a positive control [52,53,54,55]. Following inoculation, the plates were incubated without shaking at 37 °C for 24, 48, 72, and 96 h to allow the formation of a biofilm at the bottom of the wells as well as to determine the time point at which the maximum formation of biofilm occurred. After this period, the supernatants were removed, and the biofilms were washed (×3) with sterile distilled water. The biofilms were then stained with 1% crystal violet for 5 min, and further washed (×3) with tap water. A de-staining step was performed using a 7:3 (*v*/*v*) mixture of ethanol and acetone and the OD of the suspension was measured at 550 nm. The percentage of biofilm inhibition was calculated as: (OD_control_ − OD_sample_) × 100/OD_control_.

### 4.4. Molecular Docking Experiment

#### 4.4.1. Protein Preparation

BIOVIA Discovery Studio Visualizer v.4.5 (Accelrys) was used to remove all water molecules and hetero-atoms from the three-dimensional crystal structure of the *Pa*DsbA1 protein (PDB ID:5DCH) which was retrieved, complexed with its ligand inhibitor (MIPS-0000851), from the RCSB Protein Data Bank (http://www.pdb.org). AutoDock Tools v. 1.5.6rc3 was subsequently used to prepare a PDBQT file of the target protein containing added polar hydrogen atoms [62].

#### 4.4.2. Ligand Preparation

Flavonoids, phenolic acids, and other miscellaneous compounds previously reported in Manuka and/or Heather honeys [35,37,58,59,63,64,65,66,67,68,69,70,71,72,73,74] were selected as ligands for the docking experiment. All chemical structures were retrieved from SciFinder (https://scifinder.cas.org/scifinder/login). Each structure was exported to ChemOffice v.16.0 and geometry-optimised using MM2 energy minimization [75]. The structure of the ligand inhibitor (MIPS-0000851) was obtained from the RCSB Protein Data Bank (http://www.pdb.org). Docking files for all ligands were prepared using AutoDock Tools v. 1.5.6rc3 [62]. Rigid docking was performed to minimise standard errors likely due to ligands with many active rotatable bonds [76]. Gasteiger charges were assigned [77] and the files were saved as PDBQT formats in preparation for docking.

#### 4.4.3. Grid Box Preparation and Docking Studies

The size of the searching space around the *Pa*DsbA1 binding site residues was defined with grid box parameters prepared using AutoDock Tools v. 1.5.6rc3, and the molecular docking was done with AutoDock Vina v. 1.1.2 [76]. The centre of the grid box was set to x = 21.7657, y = 35.6275, z = −2.0723. Its size was 22 × 22 × 22 points in the x, y, z dimensions. The spacing was set at 1 Å. MIPS-0000851, a known *Pa*DsbA1 inhibitor, was retrieved from its co-crystallised complex with the target protein and re-docked as a control against the enzyme to validate the docking conditions. Different orientations of the ligands were searched and ranked based on their energy scores. Upon visual inspection of all binding poses obtained, only poses with the lowest root mean square deviation (RMSD) value (threshold < 1.00 Å) were considered to provide a high accuracy of docking. The default values set in Autodock Vina were used as the parameters for the rigid-ligand docking (exhaustiveness = 8). The docking scores were calculated as the predicted free energies of binding (ΔG in kcal/mol). Ligand efficiencies were calculated as the ratio of ΔG to the number of heavy atoms (NHA) for each ligand (LE = –(ΔG)/NHA) [78] (Table 1 and Table S1).

#### 4.4.4. Protein-Ligand Interactions and Predictive Inhibition

BIOVIA Discovery Studio Visualizer v.4.5 (Accelrys) was used to identify the H-bonds and non-bonding interactions between the ligand docking poses and the binding site of *Pa*DsbA1 (Table 1 and Supplementary Materials Table S1).

## 5. Conclusions

We have shown that benzoic acid (in Manuka and Heather honey) and other small molecules including MGO (in Manuka honey) have the potential to target virulence in *P. aeruginosa*. Further investigations should aim to analyse the effect of Heather honey on *P. aeruginos*a biofilm morphology using confocal laser scanning microscopy and identify the nature of its active constituent(s). Additional molecular docking could be performed to establish whether binding of honey constituents occurs in other DsbA enzymes that share a similarity with *Pa*DsbA1 (e.g., *Acinetobacter baumannii* AbDsbA). As Manuka and Heather honeys display bactericidal activity against *P. aeruginosa* [79], they represent an interesting alternative/complementary option to treat persistent wounds infected with this pathogen.

## Figures and Tables

**Table 1:** Molecular interactions of Manuka (M)/Heather (H) honey constituents showing the best ligand efficiencies for *Pa*DsbA1 ^1^.

Ligand (Origin)	Docking Score (kcal/mol)	Ligand Efficiency	Interacting Residues	Distance (Å)	Category	Type
Benzoic acid (M, H)	−5.4	0.60	Tyr148	2.408	H-Bond	Conventional
			Pro20	1.778	H-Bond	Conventional
			Leu144	3.456	Hydrophobic	Pi-Sigma
			Val30	5.365	Hydrophobic	Pi-Alkyl
			Val61	4.581	Hydrophobic	Pi-Alkyl
			Leu63	4.592	Hydrophobic	Pi-Alkyl
5-methyl-3-furan-carboxylic acid (M)	−5.1	0.57	Tyr148	2.414	H-Bond	Conventional
			Pro20	1.953	H-Bond	Conventional
			Val61	4.650	Hydrophobic	Alkyl
			Leu63	4.838	Hydrophobic	Alkyl
			Leu144	4.654	Hydrophobic	Alkyl
			Val61	4.713	Hydrophobic	Alkyl
			Leu63	4.688	Hydrophobic	Alkyl
Methyl-glyoxal (M)	−2.8	0.56	Ser22	2.996	H-Bond	Conventional
			Ser22	3.512	H-Bond	Carbon H-Bond
			Ser22	3.509	H-Bond	Carbon H-Bond
5-hydroxy-methyl-2-furaldehyde (M)	−5.0	0.56	Tyr148	2.343	H-Bond	Conventional
			Pro20	1.922	H-Bond	Conventional
			Ser22	3.508	H-Bond	Carbon H-Bond
			Ser22	3.487	H-Bond	Carbon H-Bond
			Val30	4.982	Hydrophobic	Alkyl
			Val61	4.529	Hydrophobic	Alkyl
			Leu63	4.728	Hydrophobic	Alkyl
			Leu144	4.541	Hydrophobic	Alkyl

^1^ The control had a docking score of −6.1 kcal/mol and a ligand efficiency of 0.41.

## Supplementary Materials

The following are available online at https://www.mdpi.com/2079-6382/9/12/911/s1, Figure S1: Time course assay for the determination of the optimal bacterial biofilm formation time, Table S1: Docking scores and ligand efficiencies of Manuka and/or Heather honey constituents towards *Pa*DsbA1.
