# Supplementary material for: Antibiofilm Activity of Heather and Manuka Honeys and Antivirulence Potential of Some of Their Constituents on the DsbA1 Enzyme of Pseudomonas aeruginosa
Source: Antibiotics (Basel). 2020 Dec 15;9(12):911. doi: 10.3390/antibiotics9120911 (PMC7765399; doi:10.3390/antibiotics9120911)
Supplement: Supplementary file 1 [file antibiotics-09-00911-s001.pdf]

antibiotics

MDPI

Supplementary Material

Antibiofilm Activity of Heather and Manuka Honeys and Antivirulence Potential of Some of Their Constituents on the DsbA1 Enzyme of Pseudomonas aeruginosa

Oscar Shirlaw [1,†], Zara Billah [1,†], Baraa Attar [1], Lisa Hughes [1], Rana M. Qasaymeh [1], Veronique Seidel [1,*] and Georgios Efthimiou [2,*]

[1] Strathclyde Institute of Pharmacy and Biomedical Sciences, University of Strathclyde, Glasgow G4 0RE, UK; oscar.shirlaw.2016@uni.strath.ac.uk (O.S.); zara_billah@hotmail.co.uk (Z.B.); baraa@me.com (B.A.); lisahughes6217@gmail.com (L.H.); rana-mohammad-mahmoud-qasaymeh@strath.ac.uk (R.M.Q.)

[2] Department of Biomedical and Forensic Sciences, Hardy Building, University of Hull, Hull HU6 7RX, UK; g.efthimiou@hull.ac.uk

* Correspondence: veronique.seidel@strath.ac.uk (V.S.); g.efthimiou@hull.ac.uk (G.E.)

† Please provide the explanation of the symbol.

Received: 29 September 2020; Accepted: 10 December 2020; Published: 15 December 2020

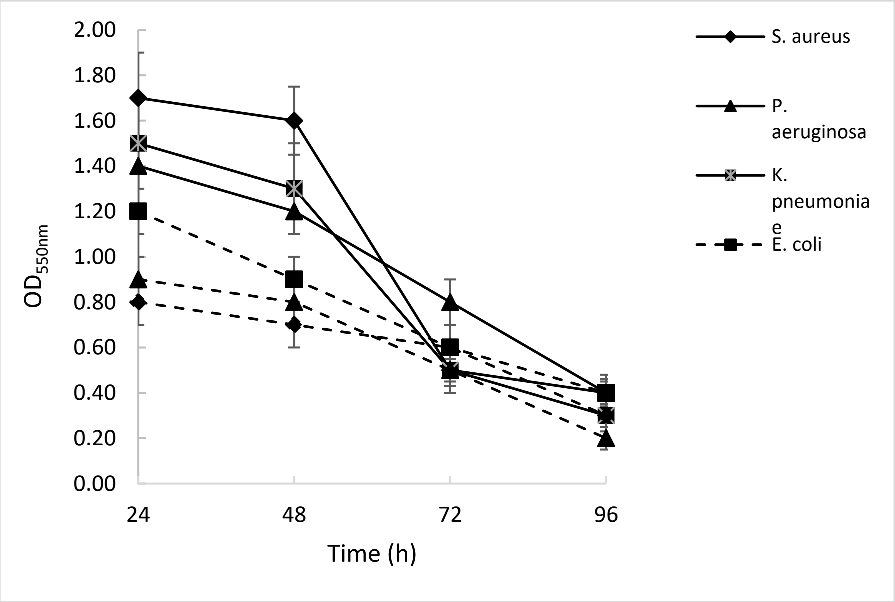

Figure S1. Time course assay for the determination of the optimal bacterial biofilm formation time.

Table S1. Docking scores and ligand efficiencies of Manuka/heather honey constituents towards *Pa*DsbA1 [1].

Origin	Compound	Docking score (kcal/mol)	Ligand efficiency (kcal/mol/NHA)
Manuka	*trans*-cinnamic acid	−6	0.55
Manuka	4-Hydroxybenzoic Acid	−5.4	0.54
Manuka	Gallic acid	−6.1	0.51
Heather	*p*-coumaric acid	−6.1	0.51
Heather	Phenylacetic Acid	−5	0.50
Manuka, heather	Caffeic acid	−6.5	0.50
Manuka	2−Methoxybenzoic Acid	−5.4	0.49
Manuka	Kojic acid	−4.9	0.49
Manuka	2-Acetyl-1-Pyrroline	−3.8	0.48
Heather	Ferulic acid	−6.3	0.45
Manuka	2'-Methoxyacetophenone	−4.8	0.44
Manuka	Chrysin	−8.3	0.44
Manuka	2,3-dimethoxynaphthalene	−6.1	0.44
Manuka	4-Methoxyphenyl acetic acid	−5.2	0.43
Manuka	Tectochrysin	−8.5	0.43
Heather	Apigenin	−8.4	0.42
Heather	Kaempferol	−8.8	0.42
Manuka	4,4'-Dimethoxystilbene	−7.6	0.42
Manuka	Syringic acid	−5.7	0.41
Manuka, heather	*trans, trans*-abscisic acid	−7.7	0.41
Manuka	Galangin	−8.1	0.41
Heather	Luteolin	−8.6	0.41
Heather	Quercetin	−9.1	0.41
Manuka	Myricetin	−9.4	0.41
Manuka	1,6,7-Trimethyllumazine	−6.2	0.41
Manuka	Quercetin 3-Methyl Ether	−9.2	0.40
Manuka, heather	Dehydrovomifoliol	−6.3	0.39
Manuka	Kaempferol 8-Methyl Ether	−9	0.39
Manuka	Lepteridine	−5.8	0.39
Manuka	3,4,5-Trimethoxybenzoic acid	−5.7	0.38
Manuka	Ellagic acid	−8.4	0.38

Heather	*cis, trans*-abscisic acid	−7.3	0.38
Manuka	Pinocembrin	−7.2	0.38
Manuka	Pinobanksin	−7.5	0.38
Manuka	3,3,4,5,5,8-hexamethyl-2,3,5,6-tetrahydro-s-indacene-1,7-dione	−7.6	0.38
Manuka	Naringenin	−7.4	0.37
Manuka	Catechin	−7.8	0.37
Manuka	Isorhamnetin	−8.5	0.37
Manuka	Desoxyanisoin	−7.1	0.37
Manuka	Methyl syringate	−5.4	0.36
Manuka	Unedone	−6.2	0.36
Manuka	2,6-dimethoxybenzoic acid benzyl ester	−7.2	0.36
Manuka	Methyl 3,4,5-trimethoxybenzoate	−5.6	0.35
Manuka, heather	Chlorogenic acid	−8.8	0.35
Heather	Hesperitin	−7.8	0.35
Manuka	Quercetin 3,3′-Dimethyl Ether	−8.5	0.35
Manuka	Tutin	−7.4	0.35
Manuka	1,5-bis(4-methoxyphenyl)-pentane-1,5-dione	−7.7	0.33
Manuka	Rotenone	−8.3	0.29
Manuka	Leptosin	−9.1	0.28
Manuka	Leptosperin	−8.7	0.24

[1] The control had a docking score of −6.1 kcal/mol and a ligand efficiency of 0.41.
